# The effects of time-restricted feeding on early phases of carcinogenesis in rat liver and colon

**DOI:** 10.3389/fnut.2026.1650934

**Published:** 2026-01-23

**Authors:** Nadia Malakmahmoudi, Roberta Pisu, Andrea Barbarossa, Francesca Pisu, Giuseppe S. Porcu, Ezio Laconi, Andrea Perra, Sergio Uzzau, Fabio Marongiu

**Affiliations:** 1 Department of Biomedical Sciences, University of Cagliari, Cagliari, Italy; 2 Department of Histopathology, "A. Businco" Oncology Hospital, Azienda Ospedaliera Brotzu, Cagliari, Italy; 3 Department of Biomedical Sciences, University of Sassari, Sassari, Italy; 4 Unit of Microbiology and Virology, University Hospital of Sassari, Sassari, Italy

**Keywords:** time-restricted feeding, early carcinogenesis, high-fat diet, liver, colon

## Abstract

**Background and aims:**

High-fat diets are established contributors to carcinogenesis through mechanisms involving altered lipid metabolism, metabolic stress, and chronic inflammation. Time-restricted feeding (TRF) has emerged as a promising non-pharmacological strategy to improve metabolic health and potentially mitigate cancer risk by optimizing glycemic control, lipid profile, and inflammatory triggers. Despite these potential benefits, the direct impact of TRF on early-stage carcinogenesis, particularly in the context of obesogenic dietary conditions, remains inadequately explored. This study aimed to investigate the effects of TRF on the development of preneoplastic lesions in the liver and colon in male and female rats exposed to high-fat (HFD) and low-fat (LFD) diets.

**Methods:**

Carcinogenesis was initiated using diethyl nitrosamine (DENA) for the liver and azoxymethane (AOM) for the colon. Rats were assigned to ad libitum feeding (AdL) or TRF (8-h feeding window) following carcinogen exposure and were euthanized at 6 or 9 months for assessment. Pre-neoplastic lesions in the liver were evaluated by glutathione S-transferase placental form (GSTP)-positive staining, while aberrant crypt foci (ACF) were analyzed in the colon. Additionally, hepatic steatosis and metabolic parameters were assessed to determine the impact of TRF.

**Results:**

No differences were observed in the incidence, size, or distribution of GSTP-positive lesions in the liver or ACF in the colon between TRF and AdL groups. TRF also failed to reduce hepatic steatosis or improve serum lipid profiles in animals fed an HFD.

**Conclusion:**

Our findings indicate that TRF does not significantly alter the development of early preneoplastic lesions in the liver or colon, regardless of dietary fat content, and further suggest that this dietary regimen is insufficient alone to counteract metabolic dysfunctions induced by highly obesogenic diets in rats.

## Introduction

Obesogenic diets are increasingly recognized as key contributors to cancer development through their profound impact on metabolic homeostasis and cellular processes (1, 2). Excessive dietary fat intake promotes obesity, a condition characterized by chronic low-grade inflammation, which plays a pivotal role in tumorigenesis (3). Adipose tissue in obese individuals secretes pro-inflammatory cytokines such as interleukin-6 (IL-6) and tumor necrosis factor-alpha (TNF-*α*), which activate signaling pathways like NF-κB and STAT3, fostering an environment conducive to cancer development (4, 5). Furthermore, high-fat diets drive lipid accumulation and insulin resistance, exacerbating hyperinsulinemia and increased levels of insulin-like growth factor-1 (IGF-1), both of which are known to enhance cell proliferation and inhibit clearance of pre-malignant cells (6, 7). These mechanisms have been linked to an elevated risk of several malignancies, including liver and colorectal (8, 9). In addition, liver lipid accumulation fuels progression of metabolic dysfunction-associated steatotic liver disease (MASLD), triggering lipotoxicity, oxidative stress, and chronic inflammation—which are key drivers of liver damage and carcinogenesis (3, 4, 10). Pro-inflammatory cytokines, such as TNF-*α* and IL-6, released during MAFLD progression, contribute to fibrosis and cirrhosis, further creating a favorable environment for HCC development (10, 11). These mechanisms underscore the significant role of high-fat diets in the pathogenesis of MAFLD and its progression to HCC (12). Addressing the carcinogenic potential of high-fat diets is essential for developing effective dietary strategies to mitigate cancer risk, especially given the global rise in obesity rates (13). Dietary interventions aim to enhance health, manage diseases, and prevent chronic conditions by modifying food intake patterns, nutrient composition, and eating behaviors (14). These strategies for instance could be focusing on meal timing, such as time-restricted eating/feeding. TRE, a widely adopted intermittent fasting strategy, restricts food intake to a defined period within a 24-h day. It has garnered interest for its effectiveness in promoting weight loss and improving dietary compliance (15–17). This eating pattern has been shown to promote weight loss (18–20), improve insulin sensitivity (21), reduce inflammation (22, 23), and lower the risk of metabolic diseases such as type 2 diabetes and cardiovascular disease. These health benefits are partly attributed to the extended fasting period, which allows the body to enter a catabolic state, enhancing fat oxidation and reducing blood sugar levels (15). Emerging research indicates that TRE may exert anti-cancer effects by influencing metabolic health, circadian rhythms, and cellular processes such as autophagy and apoptosis (17, 24–26). Disruptions in circadian rhythms have been associated with an increased risk of cancers, particularly hormone-related types like breast and prostate cancer. By aligning eating patterns with the body’s natural circadian rhythms, TRE may help mitigate this risk (27, 28).

However, despite its promising role in modulating metabolic pathways and influencing systemic health, research specifically investigating its effects on cancer development remains limited. There is a paucity of pre-clinical studies that evaluate the impact of TRF on the early stages of carcinogenesis, a critical phase where interventions may yield significant preventive benefits (29). This study aimed to address this gap by exploring the effects of TRF on the development of early pre-neoplastic lesions in well-characterized animal models. We utilized two models of chemically induced colorectal or liver carcinogenesis in rats fed a high-fat diet, which is a known tumor promoter for both tissues (30, 31). After up to 9 months of follow-up, no significant differences were observed in the incidence of liver foci/nodules or colon aberrant crypts/polyps between groups fed *ad libitum* and those on a TRF schedule. Furthermore, TRF was unable to exert any beneficial effect on lipid metabolic parameters in either liver of serum.

## Materials and methods

### Animals, housing and diet

All experiments were performed on a colony of male and female Fischer F344 rats purchased from Charles River Laboratories (Calco, Italy). All rats were maintained on an inverted alternating 12-h light/dark cycle, in a temperature and humidity-controlled environment, with water and food (chow diet from Caipet, Italy) available *ad libitum*, and housed four percage. They received humane care according to the criteria outlined in the National and European Guidelines. Animal studies were reviewed and approved by the Committee for Animal Wellbeing (Organismo Preposto al Benessere Animale, OPBA) of the University of Cagliari and the Italian Ministry of Health (aut. Nos. 121/2020-PR and 704/2020-PR). Following a one-week acclimatization to the facility, animals were subjected to two experimental protocols. One group of 28 males and 24 females was treated with 2 intraperitoneal (IP) injections of AOM at a dose of 15 mg/kg body weight, 1 week apart. A second group of 64 males and 64 females received a single IP injection of DENA at 200 mg/kg body weight. Both protocols are widely used in the literature to initiate colon or liver carcinogenesis, respectively (32–35). Four weeks after the first IP injection, for each experimental protocol, animals were subdivided into two groups, one fed with a low-fat diet (LFD) (Mucedola Srl, Italy, Cat. #PF20053) and one fed with a high-fat diet (HFD, 42% fat) (Mucedola Srl, Italy, Cat. #PF4462). The nutrient composition for both diets is reported in Supplementary Table 1. For each diet, animals were further subdivided into two different feeding regimen groups: *ad libitum* (AdL) feeding or TRF. In the AdL group, food was provided without restriction throughout the 24 h, while the TRF group was restricted to an 8-h feeding window from 08:00 to 16:00 daily. Given the inverted dark/light cycle, this corresponded to the dark, active phase. Body weight and food consumption were recorded from the start of the feeding regimens (week 0) until final sacrifice (week 34). Animals were euthanized using isoflurane anesthesia at different time points: in the liver carcinogenesis experiment, one group (24 males and 24 female) was euthanized at 6 months and the second (40 males and 40 females) at 9 months after IP injection of DENA; in the colon carcinogenesis experiment, all animals were euthanized at 9 months after the first IP injection of AOM. Blood samples were collected, and serum was separated and frozen at −80 °C immediately after collection. Liver and colon tissues were excised, and part of each tissue was either rapidly frozen for cryostat sections or fixed in 10% buffered formalin and embedded in paraffin. An age- and sex-matched group of animals fed a standard chow diet was used as a control in selected analyses.

### Histological analyses

#### Standard histology

Liver and colon tissue histology was observed on formalin-fixed/paraffin-embedded tissue sections after standard Hematoxylin and Eosin (H&E) staining. Hepatocellular steatosis was measured by Oil Red O staining on 8 μm cryosections of liver tissue. Aberrant crypt foci (ACF) in colon tissues were visualized using methylene blue staining (0.2% in ethanol) on formalin-fixed whole-mount colons.

#### Immunohistochemistry/immunofluorescence

Immunohistochemistry was performed on formalin-fixed/paraffin-embedded liver sections to detect glutathione S-transferase placental form (GST-P) expression using a commercially available kit (Rabbit Specific HRP/DAB, ab64261, Abcam, Cambridge, UK) following manufacturer’s instructions. Five μm sections were deparaffinized, rehydrated, and subjected to antigen retrieval with Sodium Citrate Buffer (10 mM Sodium Citrate, 0.05% Tween 20, pH 6.0). A primary antibody against rat GST-P (dilution 1:500, cat. no. 311, MBL International, USA) was incubated for 2 h at room temperature. Indirect staining was completed using a commercially available kit (Rabbit Specific HRP/DAB, ab64261, Abcam, Cambridge, UK) following manufacturer’s instructions. To evaluate cell proliferation, double immunofluorescence staining was performed on 5 μm frozen liver tissue sections, assessing both expression of Ki-67 and GST-P. Frozen sections were fixed in 1% acetic acid in ethanol, blocked with 2.5% goat serum for 30 min. Sections were first incubated with a primary antibody against Ki-67 (Abcam, ab16667-500, 1:100 dilution) for 2 h at room temperature then incubated with a fluorescently conjugated secondary antibody (Anti-Rabbit IgG Alexa Fluor 488, Abcam, ab150114, 1:250 dilution) for 1 h at room temperature. A second round of blocking with 2.5% goat serum was then performed, followed by incubation with the primary antibody against GSTP. Sections were then incubated with a secondary antibody (Anti-Mouse IgG Alexa Fluor 555, Abcam, Ab150114, 1:250 dilution) for 1 h at room temperature. DAPI was used as a nuclear counterstain to visualize nuclei.

#### Serum analyses

Total cholesterol, triglycerides and glucose levels were measured using commercially available kits (respectively MAK045, MAK266, MAK263, all from Sigma-Aldrich, USA) according to manufacturer’s protocol. Cytokine analysis was performed using a 12-plex immunoassay (Bio-Plex Pro™ Rat Cytokine Th1/Th2 Assay #171K1002M, Bio-Rad, California, USA) which was analyzed on a Bio-Plex 200 system (Bio-Rad, California, USA).

#### Image analysis, graphical representation of results and statistical analysis

Acquired microscopic images were processed for quantitative analysis using Image-Pro Premier software (Media Cybernetics, Rockville, USA). All microscopical analyses were carried out blindly. Graphical representation of quantitative results and statistical analysis was performed using Prism 5 (GraphPad Software, La Jolla, USA). Student’s t-test analysis was performed to compare two groups of data, while 2-way ANOVA was used for multiple comparisons. Statistical differences were accepted as significant were *p <* 0.05 (*), < 0.01 (**) and < 0.001 (***).

## Results

### Studies on liver carcinogenesis

#### Food consumption and growth curves

**Figure 1:**
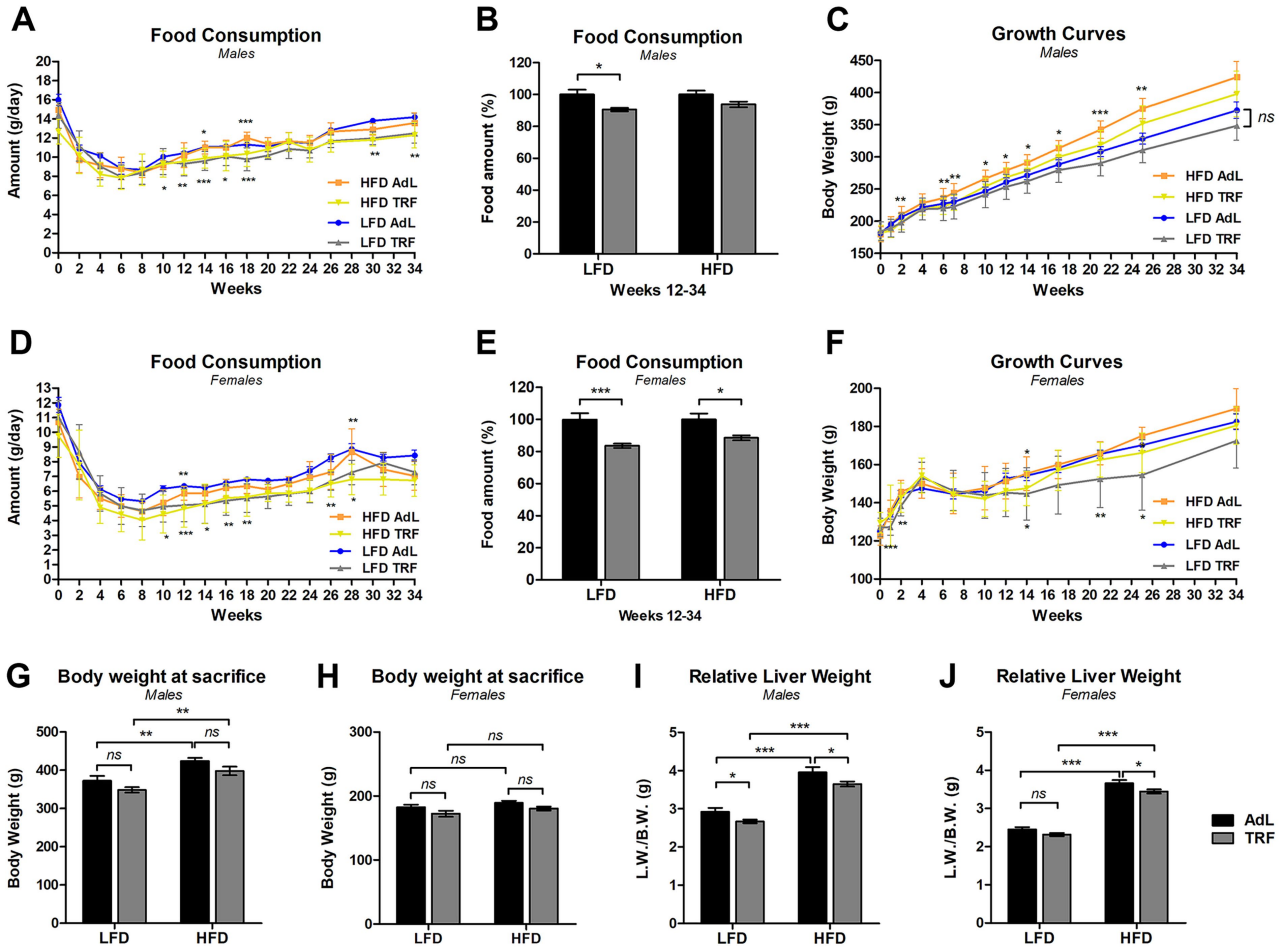
Basic biological data on male and female rats treated with DENA and subjected to different dietary and feeding regimens. Food consumption (g/rat/day) patterns in males **(A)** and females **(D)** from the start of the feeding regimens (week 0) until final sacrifice (week 34). Stars above the curves represent statistical analyses between HFD AdL and HFD TRF, while stars below the curves represent statistical analyses between LFD AdL and LFD TRF. Average food consumption over weeks 12 to 34 in males **(B)** and females **(E)**, where food intake is normalized to each AdL group, set at 100%. Growth curves of male **(C)** and female **(F)** rats from the start of the feeding regimens (week 0) until final sacrifice (week 34). Stars above the curves: HFD AdL vs. HFD TRF; stars below the curves: LFD AdL vs. LFD TRF. Body weight at the time of final sacrifice (9 months after IP injection of DENA = 34 wks post start of feeding regimens) in males **(G)** and females **(H)** rats. Relative liver weight at final sacrifice in male **(I)** and female **(J)** rats. Data are presented as mean ± standard error of the mean (SEM) (*p < 0.05, **p < 0.01, ***p < 0.001, “ns” not significant; *n =* 10 animals per group).

Food intake patterns were analyzed on rats treated with DENA, from the start of the feeding regimens (week 0) until final sacrifice (week 34) to determine whether TRF influenced overall caloric consumption. In males, all groups experienced an initial decline in food intake by week 2, followed by stabilization by week 6 (Figure 1A). From weeks 10 to 18, the LFD TRF group exhibited significantly lower food consumption compared to the LFD AdL group. In the HFD groups, significant differences in food intake between AdL and TRF were observed only at weeks 14 and 18. After week 18, the disparities in food consumption among all groups diminished. Notably, at weeks 29 and 32, the LFD AdL group again exhibited significantly higher food intake compared to the LFD TRF group. In females, the initial food intake decline was more pronounced than in males before stabilizing by week 6 (Figure 1D). Similarly to males behavior, females LFD TRF group exhibited significantly lower food consumption compared to the LFD TRF group from weeks 10 to 18 and, again, at weeks 26 and 28. In the HFD groups, as observed in males, average food intake was always higher in AdL than in TRF, reaching significant difference only at weeks 12 and 28. A comparison of food consumption as a percentage of total intake (weeks 12–34) revealed significant reductions in TRF groups compared to AdL groups in both males and females, particularly in LFD groups (*p <* 0.05) (Figures 1B,E). These findings indicate that while TRF may reduce food intake, its impact varies depending on dietary composition and sex, with more pronounced effects observed with LFD.

To assess the effects of diet and feeding regimen on body weight, growth curves were also recorded. In males, significant differences emerged based on dietary composition and feeding schedule (Figure 1C). The HFD-AdL group exhibited the highest body weights throughout the study, while HFD-TRF remained significantly lower from week 6 onward compared to HFD-AdL. In contrast, LFD AdL and LFD TRF groups showed comparable growth curves, with no significant differences between them. In females, body weight was more variable (Figure 1F), with HFD-TRF group displaying a similar growth curve compared to HFD AdL. However, LFD-TRF group showed a more consistent lower growth trend compared to LFD AdL, although a significant difference was only sporadically attained. Overall, these findings suggest that dietary fat content has a more pronounced effect on body weight than feeding regimen, particularly in females.

Analysis of body weight at sacrifice showed that male rats on a HFD had higher body weights than to their counterparts fed a LFD both at 6 months (Supplementary Figure 1A) and 9 months (Figure 1G) after IP injection of DENA (corresponding to 24 and 34 weeks after the start of the feeding regimens, respectively). At 6 months, animals in the HFD TRF group had significantly lower body weights compared to those in the HFD AdL group (Supplementary Figure 1A); however, this difference was not maintained at 9 months (Figure 1G). In female rats, body weights did not differ significantly between LFD and HFD, or between the AdL and TRF feeding regimens, suggesting that neither dietary composition nor feeding regimen had a substantial impact on body weight, at either 6 months (Supplementary Figure 1B) or 9 months (Figure 1H). This is consistent with known sex-related differences in susceptibility to diet-induced obesity, where females are generally less prone to HFD-induced weight gain, likely due to protective effects of estrogen on energy balance and lipid metabolism (36, 37).

#### Liver tissue analyses

Relative liver weight at 6 months differed significantly by diet in both male and female rats: animals on a HFD had significantly higher liver weights compared to those on a LFD, with no significant differences between AdL and TRF groups (Supplementary Figures 1 C,D). This pattern persisted at 9 months, where HFD animals continued to show significantly higher liver weights compared to their LFD counterparts (Figures 1I,J). Moreover, male animals on a TRF regimen had a reduced relative liver weight, compared to the AdL counterparts (Figure 1I). In females, HFD TRF animals had significantly lower liver weights compared to the HFD AdL group, while no significant difference was detected between LFD AdL and LFD TRF groups (Figure 1J).

**Figure 2:**
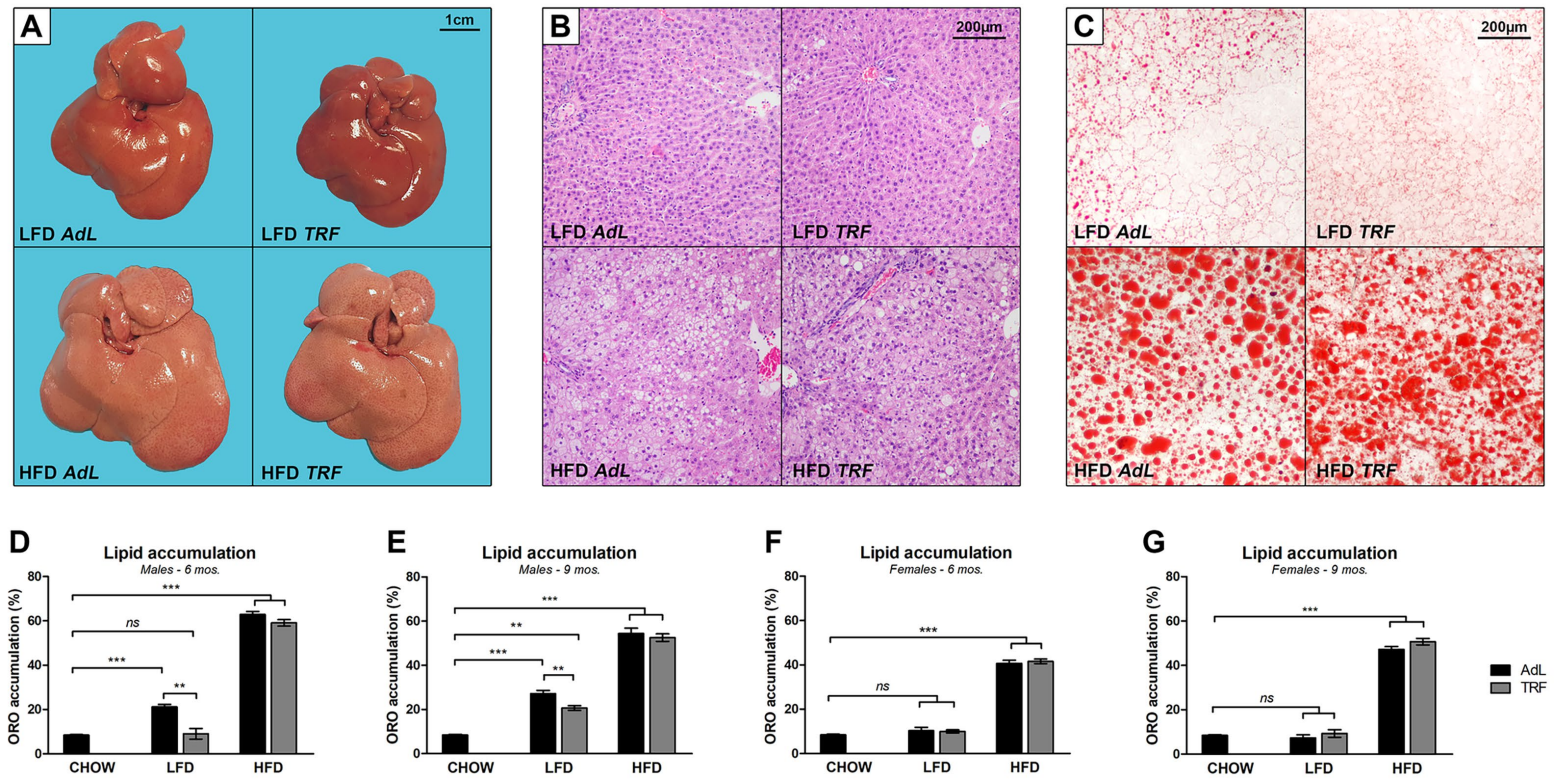
Liver tissue analyses. Representative images showing the gross appearance of liver tissues **(A)** and H&E microscopical analysis of tissue morphology **(B)** under different dietary conditions in male rats. Representative images of Oil Red O staining (ORO) of liver sections from male animals **(C)**. Scale bars shown in each panel. Quantification of lipid accumulation, assessed by ORO, in male rats sacrificed at 6 **(D)** and 9 **(E)** months after DENA injection; and in female rats sacrificed at 6 **(F)** and 9 **(G)** months after DENA injection (*n =* 6 animals per group at 6 months; *n =* 10 animals per group at 9 months). An age- and sex-matched group of animals fed a standard chow diet was used as a control (*n =* 5). Data are presented as mean ± SEM (*p < 0.05; **p < 0.01; **p < 0.001; “ns” not significant).

Under macroscopic the liver surface of LFD-fed animals (Figure 2A, top) appeared regular. Histological analysis revealed mild steatosis, characterized by occasional hepatocyte swelling and vacuolation (Figure 2B, top). In contrast, livers from HFD-fed animals were enlarged (Figure 2A, bottom) and exhibited more pronounced steatosis, as expected (Figure 2B, bottom). No noticeable differences were observed between the AdL and TRF groups. Macroscopic examination at sacrifice revealed the presence of hepatic nodules across dietary groups, with the most prominent observed in HFD-fed males, where four animals developed nodules, the largest measuring 1 cm with vascularization. In HFD-fed females, the largest nodule measured 4 mm in diameter.

Quantitative analysis of hepatic lipid content (Figures 2D–G) confirmed a sizeable increase in lipid accumulation in both male and female rats fed a HFD, compared to those on a LFD or standard chow, at both 6 and 9 months. Notably, no significant differences were observed between AdL and TRF within the HFD groups at any time point or in either sex, indicating that TRF does not mitigate HFD-induced hepatic lipid accumulation. Interestingly, in males only, LFD-AdL animals exhibited significantly higher lipid accumulation compared to chow-fed controls at both 6 and 9 months (Figures 2D,E). This effect was attenuated by TRF, suggesting a protective effect of TRF against lipid accumulation under LFD feeding in males (>50% lower at 6 months, *p* = 0.0011; >20% lower at 9 months, *p* = 0.0026).

#### Systemic profiles of metabolic and inflammatory markers

**Figure 3:**
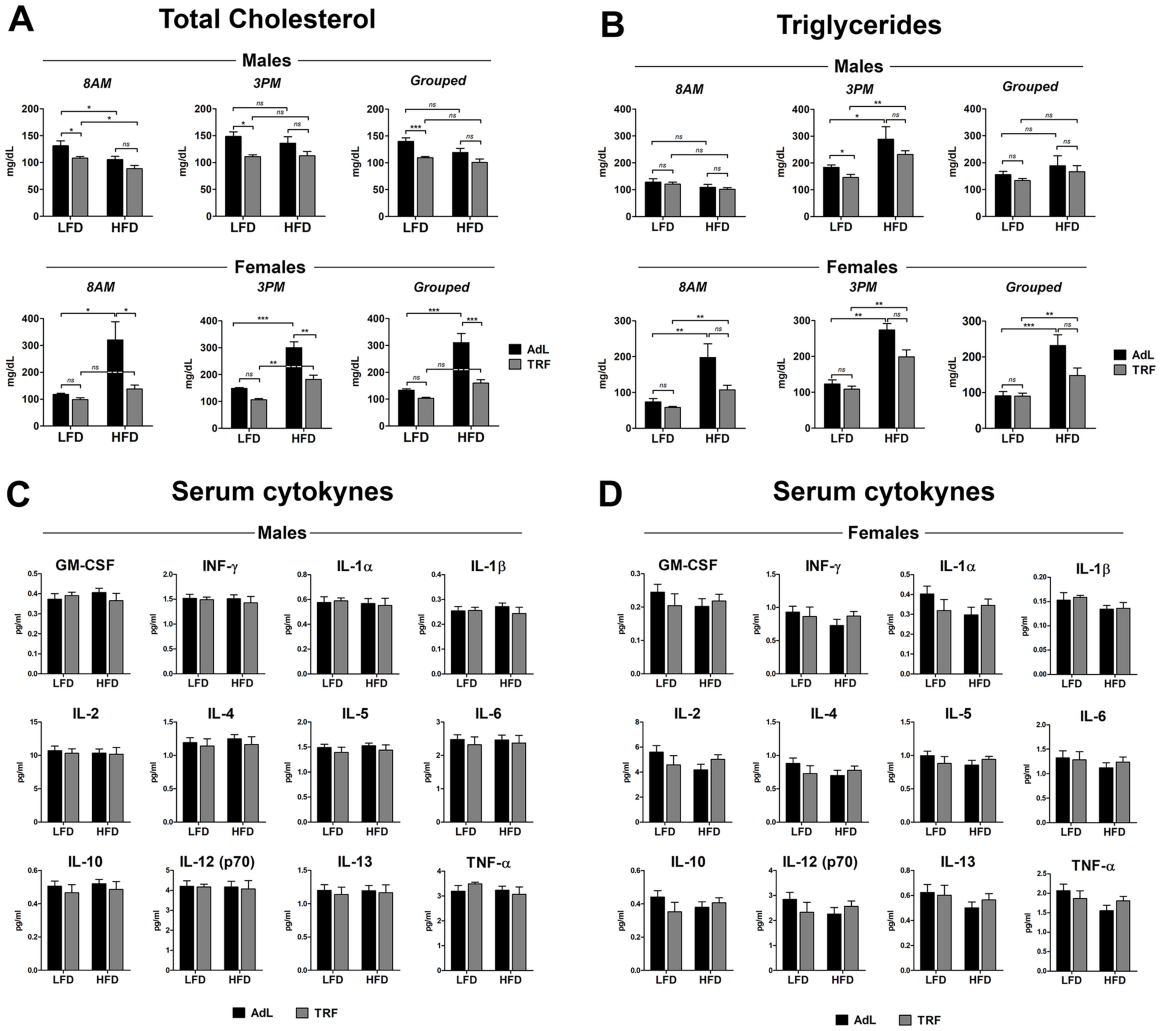
Effect of dietary interventions on systemic profiles of metabolic and inflammatory markers at 9 months after IP injection of DENA. Serum concentration of total cholesterol **(A)** and triglycerides **(B)** from rats sacrificed at 8 a.m. (during the fasting hours for TRF) and 3 p.m. (during the active and feeding phase) or shown as a combined (grouped) analysis. Serum concentration of a panel of circulating cytokines in male **(C)** and female **(D)** animals. Data are presented as mean ± SEM. (*p < 0.05, **p < 0.01, ***p < 0.001; *n =* 10 animals per group). For C and D, absence of asterisks denotes non-significant differences.

Serum analyses were conducted on samples collected at the time of final sacrifice (9 months after IP injection of DENA) at two time points: 8 a.m. (at the end of the fasting phase for TRF groups) and 3 p.m. (during the active or feeding phase). Total cholesterol measurements (Figure 3A) indicated that, in males, TRF significantly reduced cholesterol levels in the LFD group at both time points. Grouped analysis (including both 8 a.m. and 3 p.m. samples) confirmed a consistent reduction in cholesterol levels with TRF under LFD, while no significant differences were observed between TRF and AdL in the HFD-fed group. In females, HFD AdL-fed animals exhibited significantly elevated total cholesterol levels, which were significantly reduced in the HFD TRF group, whereas no effect was observed between LFD AdL and LFD TRF groups.

Triglyceride levels showed a significant increase in HFD-fed male animals compared to those on LFD, both on AdL and on TRF regimens, but this effect was limited to the active or feeding phase (Figure 3B). TRF led to a partial reduction of serum triglycerides, reaching statistical significance only in the LFD group. No significant differences were observed between diet or feeding regimens during the fasting hours or in the combined (grouped) analysis. In females, a similar trend was observed where HFD-fed animals exhibited higher triglyceride levels, and TRF appeared to attenuate this increase, although the reduction did not reach statistical significance (Figure 3B). This pattern held true also during the fasting hours and the combined analysis.

Serum glucose concentrations were also measured in male and female rats at the 9 months’ time point (Supplementary Figure 2). In males, glucose levels measured at 8 a.m. (Panel A) and 8 p.m. (Panel B) showed no significant differences between AdL and TRF groups, regardless of dietary condition (LFD or HFD). In females, glucose levels measured at 8 a.m. (Panel C) also showed no significant differences between AdL and TRF for either LFD or HFD. However, at 8 p.m. (Panel D), HFD AdL animals exhibited an increase in glucose levels that was significantly reduced in HFD TRF animals.

A comprehensive panel of circulating cytokines was measured in the serum of male (Figure 3C) and female (Figure 3D) rats collected at 9 months following IP injection of DENA. No significant differences were detected in either sex as a result of diet or feeding regimen.

#### Incidence of early pre-neoplastic lesions in the liver

**Figure 4:**
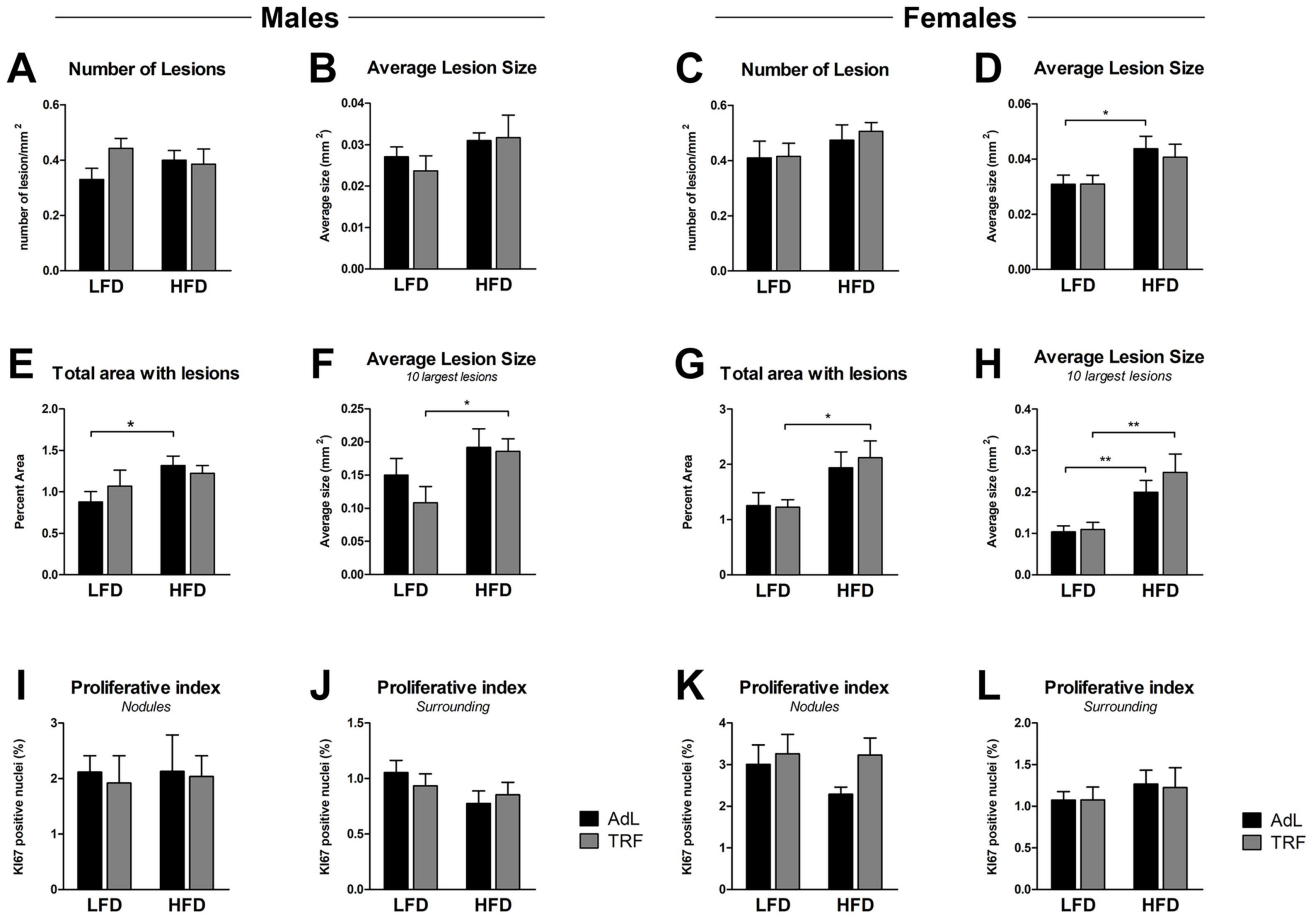
Quantitative characterization of liver lesion in male and female animals euthanized at 9 months after initiation with DENA. The number of lesions per area **(A,C)**, the average lesion size per group **(B,D)**, the total area affected by lesions **(E,G)**, and the average size of the 10 largest lesions **(F,H)** are displayed for both LFD and HFD groups under AdL and TRF. Proliferative index of Ki67-positive nuclei (%) in nodules **(I,K)** and surrounding liver tissue **(J,L)** of male and female animals. Data are presented as mean ± SEM (*p < 0.05, **p < 0.01; absence of asterisks denotes non-significant differences; *n =* 10 animals per group).

Early nodules were identified through the expression of the marker GST-P and through histological examination with H&E staining. At the 6-month time point, in males, neither diet nor feeding regimen significantly influenced number of lesions/sq.cm or average lesion size (Supplementary Figures 3A,B). Similarly, total lesion-affected area and the average size of the 10 largest lesions did not differ significantly across groups (Supplementary Figures 3E,F). In females, number of lesions/sq.cm and average lesion size remained unchanged across groups (Supplementary Figures 3C,D). However, the total lesion-affected area reached a significantly higher percentage in the HFD AdL group compared to LFD AdL, and the average size of the ten largest lesions was also significantly increased in the HFD AdL group, suggesting that HFD promotes lesion expansion, whereas TRF did not significantly alter lesion burden (Supplementary Figures 3G,H). At the 9-month time point, in males, the number of lesions/sq.cm and average lesion size remained similar across LFD and HFD groups, with no significant differences between AdL and TRF regimens (Figures 4A,B). However, the total lesion-affected area displayed a marginal increase in HFD AdL group compared to LFD AdL (Figure 4E). Similarly, the average size of the ten largest lesions was slightly higher in HFD TRF compared to LFD TRF (Figure 4F). In females, number of lesions/sq.cm did not differ significantly between LFD and HFD groups, indicating a comparable lesion burden across diets (Figure 4C). However, for the other three parameters, higher values were consistently recorded in HFD groups compared to LFD counterparts (Figures 4D–H).

Liver cell proliferation was assessed at the 9-month time point following DENA initiation by measuring the percentage of Ki67-positive nuclei in both the surrounding liver tissue and within nodules (Figures 4I–L). No significant differences were observed between LFD- and HFD-fed animals in either tissue compartment. Similarly, feeding regimen (AdL vs. TRF) had no discernible effect on Ki67 expression in either dietary group. These findings indicate that neither dietary fat content nor feeding pattern significantly influenced hepatic proliferative activity under the experimental conditions of our studies.

### Studies on colon carcinogenesis

Food intake patterns and growth curves were also analyzed in rats treated with AOM, from the start of the feeding regimens (week 0) until final sacrifice (week 34) (Supplementary Figure 4). Results show no noticeable differences compared to animals treated according to the protocol for liver carcinogenesis, suggesting that the carcinogenic treatment had no evident effects on basic biological parameters.

**Figure 5:**
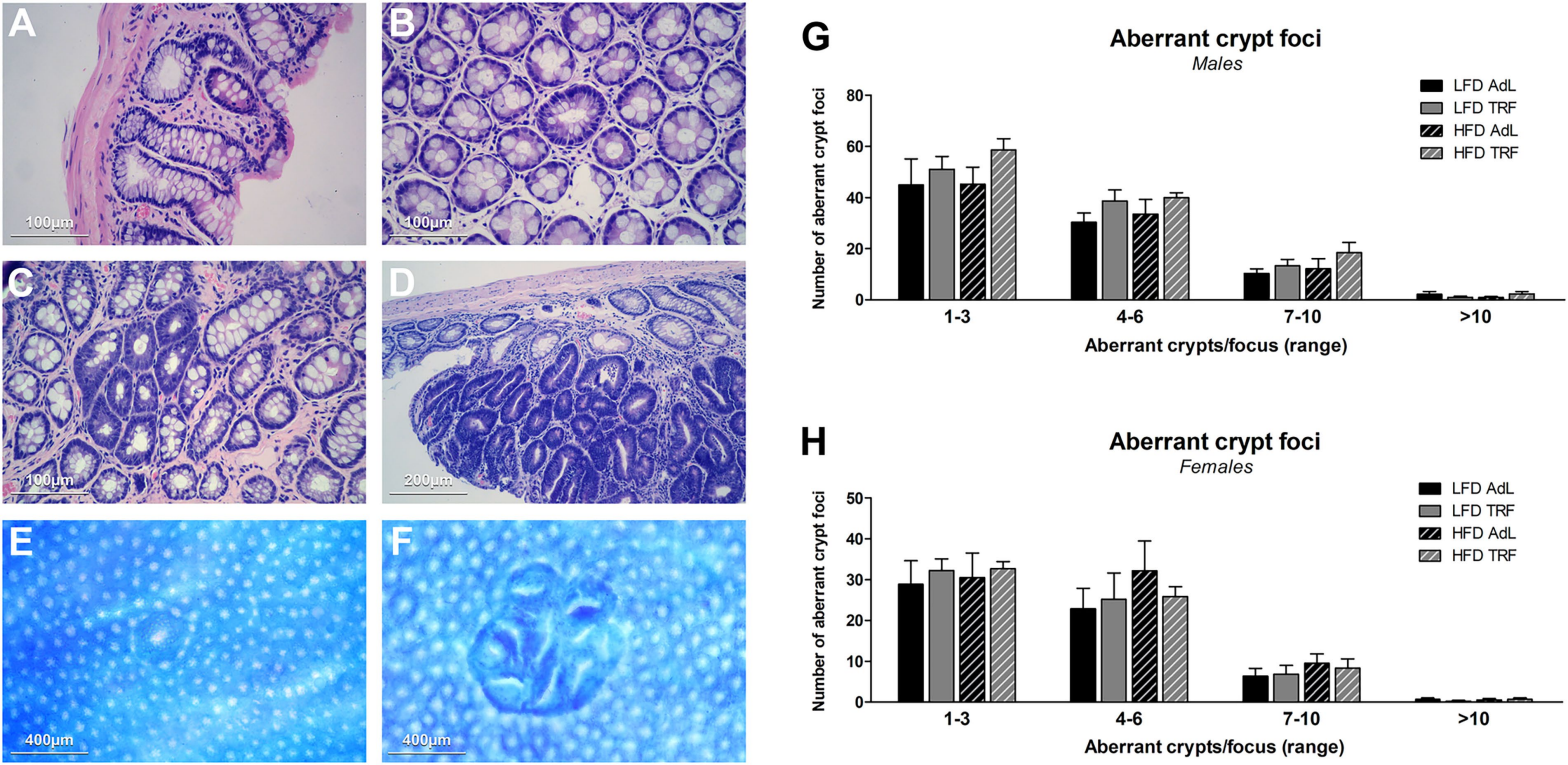
Analysis of lesions in the colon. Representative H&E-stained sections of colonic tissue. **(A)** Normal colonic crypts displaying well-organized, cylindrical structures with uniform epithelial lining and intact lamina propria. **(B)** Single polyp characterized by a mildly disorganized crypt architecture with slight dilation and loss of uniformity. **(C)** Multiple polypoid formations with distorted crypts, irregular shapes, and evidence of early hyperplastic changes, including increased nuclear-to-cytoplasmic ratios suggestive of early dysplasia. **(D)** Advanced adenoma displaying severe crypt disorganization, hyperchromatic nuclei, pseudo stratification, and glandular crowding, indicative of significant dysplastic transformation. Representative micrographs of colon whole-mounts stained with methylene blue to reveal ACF: **(E)** single focus and **(F)** multiple-foci aberrant crypts. Scale bars shown in each panel. The number of ACFs in male **(G)** and female **(H)** animals, categorized by the number of crypts per focus (from 1 to 3, 4 to 6, 7 to 10, and more than 10). Data are presented as mean ± SEM and differences among groups are not statistically significant; *n =* 6 animals per group.

Macroscopic and microscopic examination of the colons in male and female rats revealed the presence of a few polyps and adenomas ranging from 5 mm to 1 cm in size (Supplementary Table 2). Their frequency was independent of feeding regimen (AdL or TRF). Adenomas were mostly localized to the distal colon. A notable case was observed in a male animal fed HFD, where a large (3 cm) tumour mass was present, exhibiting a distinct cauliflower-like morphology. Colon tissue sections were analyzed for the identification of morphological abnormalities via H&E (Figures 5A–D).

To assess the distribution of aberrant crypt foci (ACF), lesions were categorized based on the number of crypts per focus into four groups: 1–3, 4–6, 7–10, and >10 crypts. ACF were predominantly found in the 1–3 and 4–6 categories in both male and female animals, with very few foci observed in the higher crypt number ranges (Figures 5G,H). In males, a slight increase in ACF numbers was noted in the HFD-TRF group, particularly within the smaller crypt categories, although this difference was not statistically significant. Females showed a similar distribution pattern, with no significant differences across diet or feeding regimens. Overall, neither dietary fat content nor feeding pattern had a significant effect on ACF number or size in either sex.

## Discussion

In these studies, we investigated the effect of TRF on the development of early lesions in two animal models of chemically-induced liver and colon carcinogenesis. Following initiation, male and female animals were exposed to either HFD or LFD, delivered either *ad libitum* or according to a TRF protocol. The first observation is that TRF regimen resulted in a slight (around 10%) decrease in overall food consumption, more evident in female animals. This was paralleled by a corresponding delay in growth rate between TRF and AdL groups. However, at the end of the study, no significant differences in body weight were seen between the two dietary regimens, in either male or female animals. This pattern of results was consistent in both liver and colon carcinogenesis studies and across sexes. Furthermore, TRF had only marginal or no effect on liver lipid accumulation, serum lipid parameters or cytokine markers of systemic inflammation, with female animals being even more refractory than males.

The findings reported above are in contrast with those of a series of previous investigations, which described the ameliorating effects of TRF on HFD-induced weight gain, obesity and liver steatosis (38–41). The reasons for these discrepancies are not immediately apparent. Most studies so far have been conducted in mice, while the present investigation was carried out in rats. It is therefore possible that interspecies differences may exist in the impact of TRF on overall metabolism. However, this possibility is unlikely because at least one study, conducted in rats, reports a significant reduction in body mass index associated with TRF under high fat diet (42). On the other hand, Sato and Oishi observed a limited effect of TRF on total food consumption, growth curves and hepatic lipid accumulation in a choline-deficient high-fat diet-induced model of steatohepatitis (43). Of note, these studies were performed in mice and their results are in line with our present data obtained in rats. Thus, it is unlikely that any interspecies difference between mice and rats can account for the observed discrepancies in the reported effects of TRF.

Another possibility pertains to differences in diet composition among different studies. For example, in the study by Chaix et al., the high-fat diet used contained 60% fat by energy (39), while in our present investigation the level of dietary fat was 42%. How the effect of TRF could be possibly related to the quality of diet is presently unknown. We have recently proposed that both dietary quality and quantity may impact the potential of TRF to exert its beneficial effects on overall health (44). Thus, under optimum dietary conditions TRF may have no additional benefits over a free-schedule eating pattern. This proposition is supported by investigations conducted on humans indicating that time-restricted eating did not further improve the effects of caloric restriction alone. In one study, participants in the two groups exhibited similar reductions in body weight, body fat, waist circumference, lean mass, and metabolic risk factors, such as blood glucose levels, insulin sensitivity, lipid profiles, and blood pressure (38), suggesting that the observed changes in metabolic health parameters and body composition were predominantly driven by caloric intake reduction rather than the timing of food consumption. Furthermore, TRE was unable to exert any additional benefit over caloric restriction alone on liver lipid accumulation and metabolic risk factors in obese individuals with metabolic associated fatty liver disease (45). Similar results were reported when a hypocaloric mediterranean diet was offered for 12 weeks to patients with MASLD: cardiometabolic risk factors were equally improved in both individuals who had time-unrestricted and in those with time-restricted access to food (46, 47).

On the opposite extreme, any beneficial effect of TRF might be overwhelmed and offset by diets of poor overall quality. This possibility could help explain the results of the present studies and those of Sato and Oishi referred to above (43), as well as observations derived from the TREAT interventions, in which a time-restricted eating schedule was unable to affect fat mass, lean mass, energy intake and energy expenditure compared to eating throughout the day (48). Importantly, this study involved individuals overweight or obese (BMI from 27 to 43) and no recommendations were made to the participants as to the quantity/quality of dietary intake during the 12-week period of follow-up (48).

The second relevant observation of our studies relates to the lack of effect of TRF on the development of early pre-neoplastic lesions in either liver or colon. This was true for both LFD and HFD-fed groups in both male and female animals. To our knowledge, this is the first report describing the effect of TRF on well characterized experimental models of liver and colon carcinogenesis, which are recognized to mimic closely spontaneous cancer development in humans (49, 50). A few studies have previously addressed the impact of TRF on cancer development in different contexts. Sundaram and Yan reported a delayed progression of mammary tumors in MMTV-PyMT mice, carrying the mouse mammary tumor virus long terminal repeat which drives the mammary gland-specific expression of the polyoma virus middle T antigen (51). More recently, Shi et al. observed a reduction in the incidence of urethane-induced lung adenomas in mice exposed to TRF compared to *ad libitum*-fed controls (52). Sporadic epidemiological observations also exist in humans. A restricted eating time was found to decrease the risk of breast cancer (53) while a longer interval between last meal and sleep was associated with a lower risk of breast and prostate cancer. However, our results do not support a protective role of TRF during early phases of carcinogenesis. This might be expected, given that TRF was also unable to significantly affect obesity, liver fat accumulation and serum lipid parameters under the experimental conditions of our studies.

We conclude that the TRF regimen did not significantly reduce overall growth, liver fat accumulation, or serum lipid parameters, nor did it decrease the incidence of preneoplastic lesions in the liver or colon of rats. These results highlight the complexity of the relationship between diet and cancer, which likely extends to the specific impact of TRF on a long-term process such as neoplastic disease. Further studies are required to extend these findings to other tissues and to ascertain whether they apply to later phases of the carcinogenic process.

## Data Availability

The raw data supporting the conclusions of this article will be made available by the authors, without undue reservation.

## Supplementary material

Supplementary Figure 1 Body weight at the time of first sacrifice (6 months after IP injection of DENA = 24wks post start of feeding regimens) in males (G) and females (H)rats. Relative liver weight at 6 months sacrifice in male (I) and female (J) rats. Data are presented as mean ± standard error of the mean (SEM) (**p <* 0.05, ***p <* 0.01, ****p <* 0.001, “ns” not significant; *n =* 6 animals per group).

Supplementary Figure 2 Serum glucose concentrations from male (top) and female (bottom) rats sacrificed at 8 a.m. (during the fasting hours for TRF, left panels) and 3 p.m. (during the active and feeding phase, right panels). Data are presented as mean ± SEM. (**p <* 0.05, ***p <* 0.01, ****p <* 0.001; *n =* 5 animals per group).

Supplementary Figure 3 Quantitative characterization of liver lesion in male and female animals euthanized at 6 months after initiation with DENA. The number of lesions per area **(A,C)**, the average lesion size per group **(B,D)**, the total area affected by lesions **(E,G)**, and the average size of the 10 largest lesions **(F,H)** are displayed for both male and female animals. Data are presented as mean ± SEM. (**p <* 0.05; *n =* 6 animals per group).

Supplementary Figure 4 Basic biological data on male and female rats treated with AOM and subjected to different dietary and feeding regimens. Food consumption patterns in males **(A)** and females **(B)** from the start of the feeding regimens (week 0) until final sacrifice (week 34). Stars above the curves represent statistical analyses between HFD AdL and HFD TRF, while stars below the curves represent statistical analyses between LFD AdL and LFD TRF. Growth curves of male **(C)** and female **(D)** rats from the start of the feeding regimens (week 0) until final sacrifice (week 34). Stars above the curves: HFD AdL vs. HFD TRF; stars below the curves: LFD AdL vs. LFD TRF. Body weight at the time of final sacrifice (9 months after first IP injection of AOM = 34wks post start of feeding regimens) in males **(E)** and females **(F)** rats. Data are presented as mean ± SEM (**p <* 0.05, ***p <* 0.01, ****p <* 0.001, “ns” not significant; *n =* 6 animals per group).

## Glossary

ACF: Aberrant Crypt Foci
AdL: *Ad Libitum*
AOM: Azoxymethane
DAB – 3,3′-Diaminobenzidine
DENA: Diethylnitrosamine
EGFR: Epidermal Growth Factor Receptor
GSTP: Glutathione S-Transferase Placental Form
H&E: Hematoxylin and Eosin
HCC: Hepatocellular Carcinoma
HFD: High-Fat Diet
IL-6: Interleukin-6
IP: Intraperitoneal
LFD: Low-Fat Diet
MASLD: Metabolic Dysfunction-Associated Steatotic Liver Disease
NF-κB: Nuclear Factor Kappa-Light-Chain-Enhancer of Activated B Cells
ORO: Oil Red O
STAT3: Signal Transducer and Activator of Transcription 3
TNF-*α*: Tumor Necrosis Factor-Alpha
TRE: Time-Restricted Eating
TRF: Time-Restricted Feeding
