# Supplementary figures and images for: The effects of time-restricted feeding on early phases of carcinogenesis in rat liver and colon
Source: Front Nutr. 2026 Jan 23;13:1650934. doi: 10.3389/fnut.2026.1650934 (PMC12875941; doi:10.3389/fnut.2026.1650934)

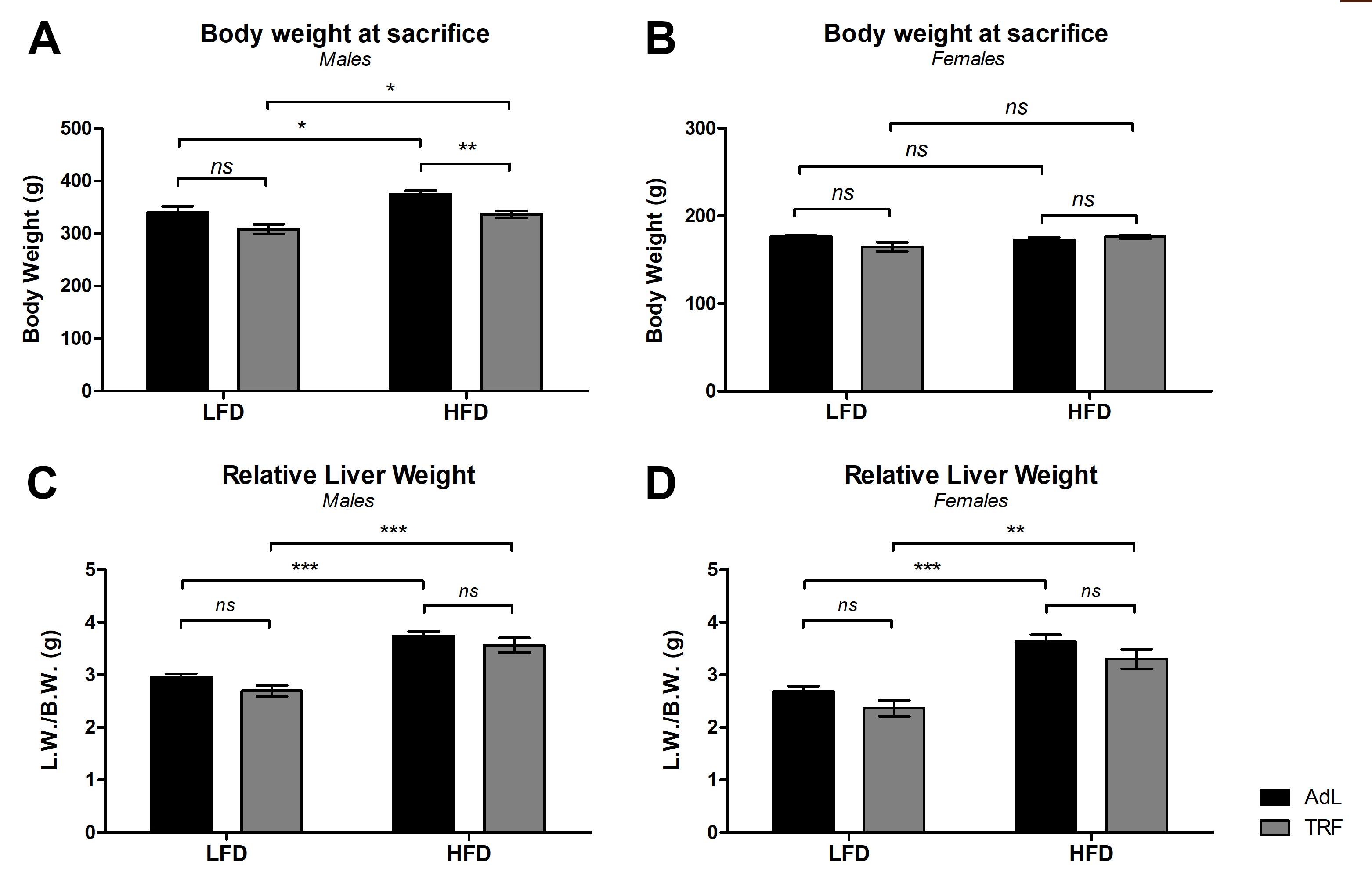

Supplement: Supplementary Figure 1 — Body weight at the time of first sacrifice (6 months after IP injection of DENA = 24wks post start of feeding regimens) in males (G) and females (H)rats. Relative liver weight at 6 months sacrifice in male (I) and female (J) rats. Data are presented as mean ± standard error of the mean (SEM) (*p < 0.05, **p < 0.01, ***p < 0.001, “ns” not significant; n = 6 animals per group). [file Image_1.tif]

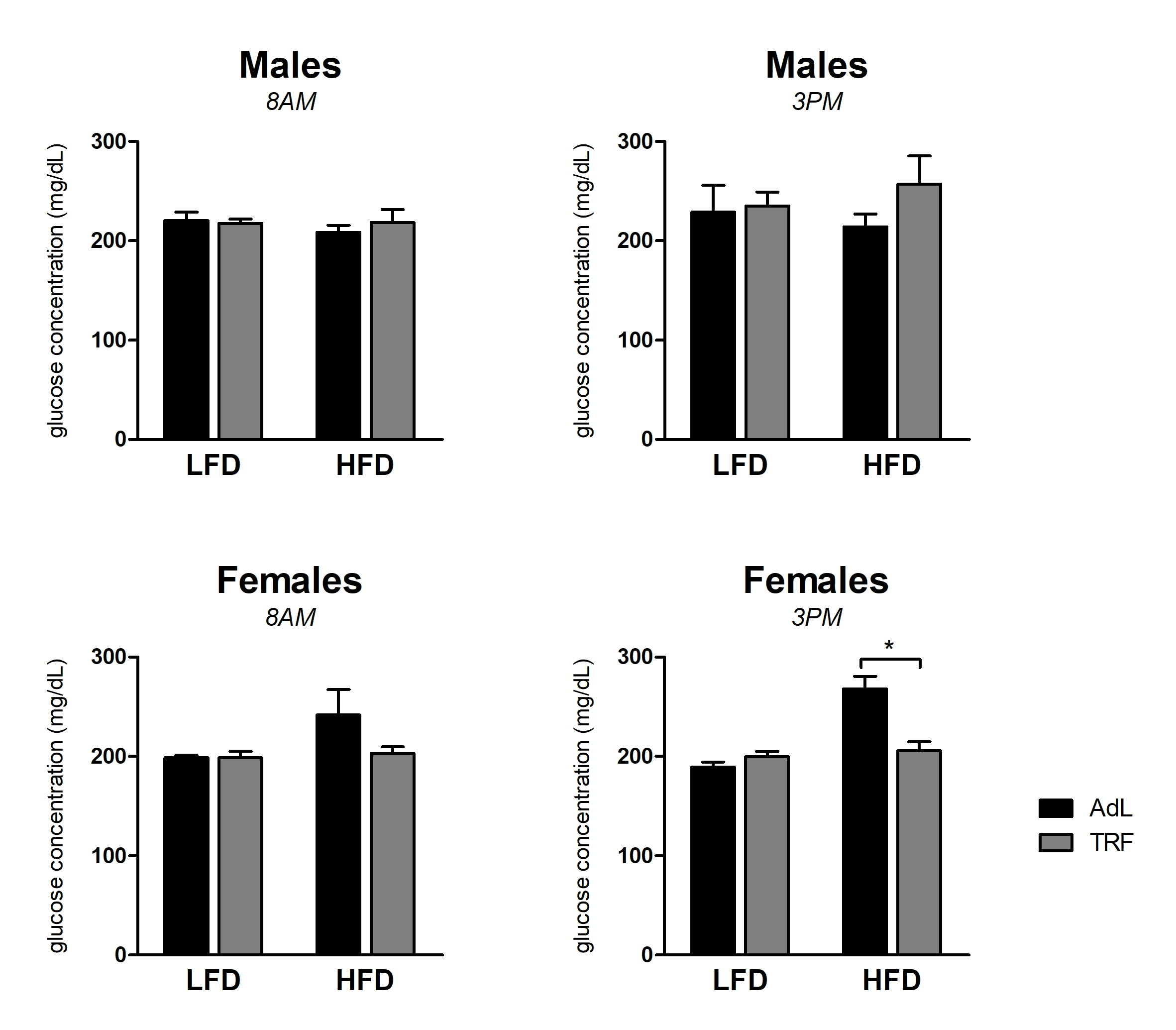

Supplement: Supplementary Figure 2 — Serum glucose concentrations from male (top) and female (bottom) rats sacrificed at 8 a.m. (during the fasting hours for TRF, left panels) and 3 p.m. (during the active and feeding phase, right panels). Data are presented as mean ± SEM. (*p < 0.05, **p < 0.01, ***p < 0.001; n = 5 animals per group). [file Image_2.tif]

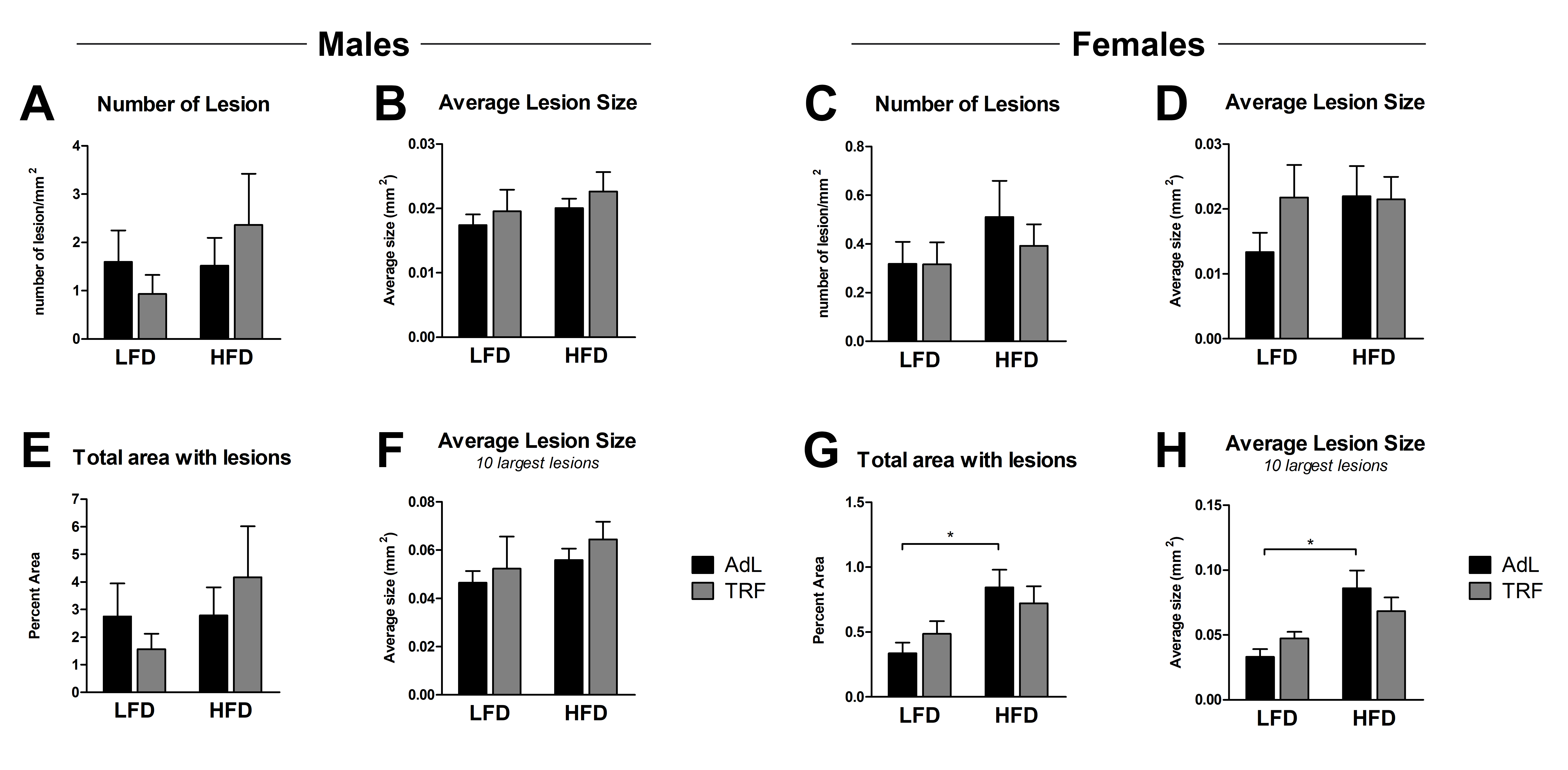

Supplement: Supplementary Figure 3 — Quantitative characterization of liver lesion in male and female animals euthanized at 6 months after initiation with DENA. The number of lesions per area (A,C), the average lesion size per group (B,D), the total area affected by lesions (E,G), and the average size of the 10 largest lesions (F,H) are displayed for both male and female animals. Data are presented as mean ± SEM. (*p < 0.05; n = 6 animals per group). [file Image_3.tif]

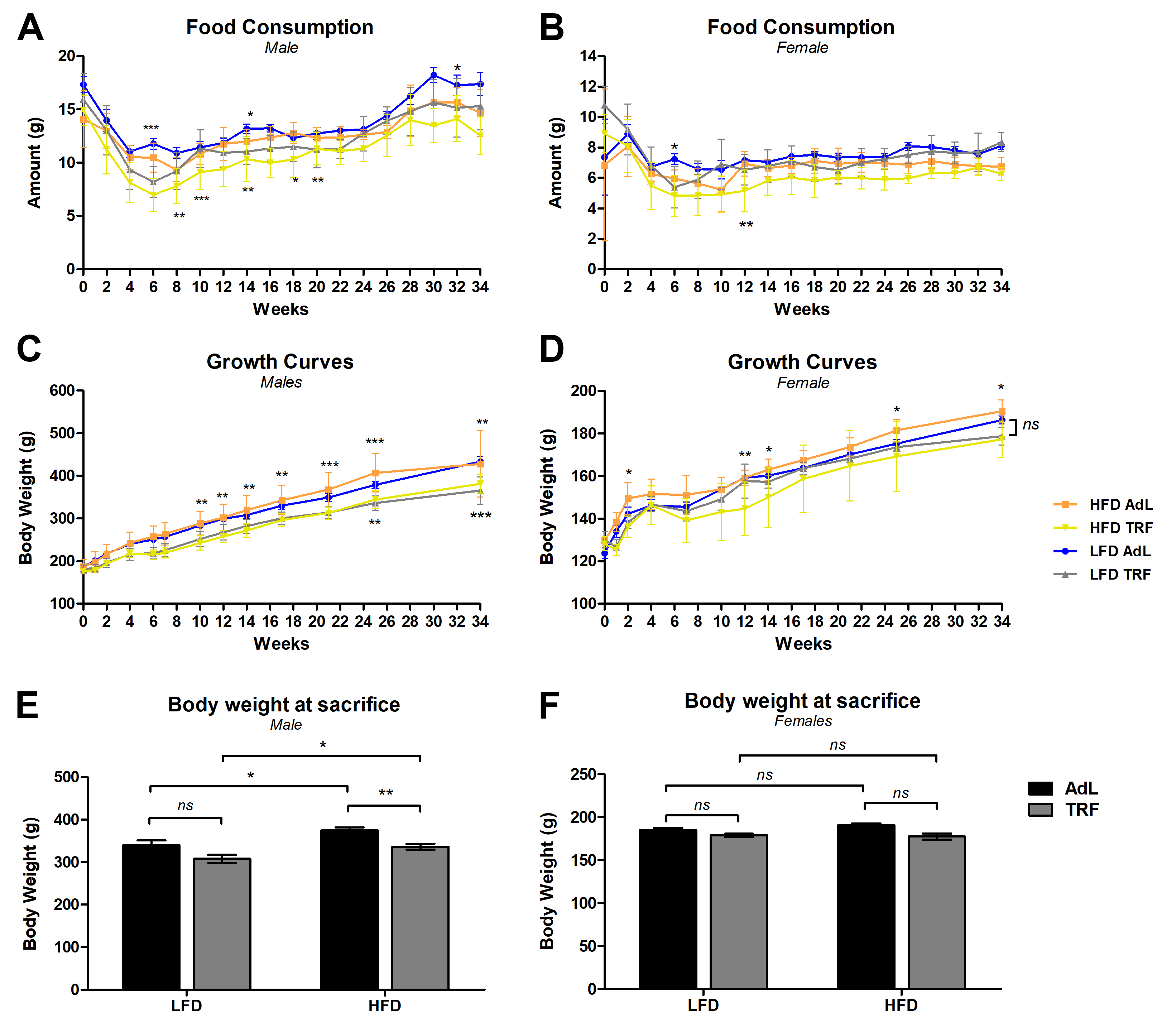

Supplement: Supplementary Figure 4 — Basic biological data on male and female rats treated with AOM and subjected to different dietary and feeding regimens. Food consumption patterns in males (A) and females (B) from the start of the feeding regimens (week 0) until final sacrifice (week 34). Stars above the curves represent statistical analyses between HFD AdL and HFD TRF, while stars below the curves represent statistical analyses between LFD AdL and LFD TRF. Growth curves of male (C) and female (D) rats from the start of the feeding regimens (week 0) until final sacrifice (week 34). Stars above the curves: HFD AdL vs. HFD TRF; stars below the curves: LFD AdL vs. LFD TRF. Body weight at the time of final sacrifice (9 months after first IP injection of AOM = 34wks post start of feeding regimens) in males (E) and females (F) rats. Data are presented as mean ± SEM (*p < 0.05, **p < 0.01, ***p < 0.001, “ns” not significant; n = 6 animals per group). [file Image_4.tif]
