# Supplementary material for: The effects of time-restricted feeding on early phases of carcinogenesis in rat liver and colon
Source: Front Nutr. 2026 Jan 23;13:1650934. doi: 10.3389/fnut.2026.1650934 (PMC12875941; doi:10.3389/fnut.2026.1650934)
Supplement: Supplementary file 5 [file Table_1.pdf]

Supplementary Table 1. Diet composition

		LFD	HFD
	**Name**	Low Fat control diet	42% kcal/Fat diet (Inc. Sucrose, 1.25% Chol)
	**Cat.no.**	PF4462	PF20053
	**Ingredients**		
**Nutritional additives (per kg)**	Vitamin A	19800 I.U.	19800 I.U.
	Vitamin D3	2200 I.U.	2200 I.U.
	Fe	40 mg	40 mg
	Mn	58.5 mg	58.5 mg
	Zn	32.5 mg	32.5 mg
	Cu	5.8 mg	5.8 mg
	I	0.21 mg	0.21 mg
	Se	0.10 mg	0.10 mg
**Analytical constituents**	Crude protein	17.50 %	17.30 %
	Crude oils and fats	5.50 %	21.20 %
	Crude fibers	3.00 %	3.00 %
	Crude ash	4.00 %	3.00 %
**Caloric Values**	Gross energy	4.0 kCal/g	4.8 kCal/g
	Protein	20.5 %	16.7 %
	Fat	12.3 %	41.9 %
	Carbohydrates	65.6 %	42.2 %

Supplementary Table 2. Total number of observed macroscopic nodules in the colon

		Males		Females	
		AdL	TRF	AdL	TRF
Colon	LFD	3	-	-	2
	HFD	4	4	-	-
